# Evaluation of Robotic Versus Laparoscopic Surgery for Right Colon Cancer Treatment: Systematic Review and Meta-Analysis

**DOI:** 10.3390/jcm15041493

**Published:** 2026-02-14

**Authors:** Martina Sardonini, Daniele Giuliani, Alessandro Spizzirri, Vincenzo Napolitano, Roberto Cirocchi, Luca Properzi, Francesco Brucchi, Sara Lauricella, Francesca Pennetti Pennella, Valentina Bartolini, Marco Coccetta

**Affiliations:** 1 Dipartimento di Medicina e Chirurgia, University of Perugia, 06132 Perugia, Italy; martina.sardonini@gmail.com (M.S.); luca.properzi@yahoo.it (L.P.); 2 Struttura di Chirurgia Generale Colo-Proctologica dell’Azienda Ospedaliera di Terni, 05100 Terni, Italy; d.giuliani@aospterni.it (D.G.); a.spizzirri@aospterni.it (A.S.); v.napolitano@aospterni.it (V.N.); f.pennettipennella@aospterni.it (F.P.P.); v.bartolini@aospterni.it (V.B.); m.coccetta@aospterni.it (M.C.); 3 Università di Milano, 20122 Milan, Italy; francesco.brucchi@unimi.it; 4 Dipartimento di Fisiopatologia e Trapianti, Università degli Studi di Milano, 20122 Milan, Italy; sara.lauricella@istitutotumori.mi.it

**Keywords:** right colectomy, robotic surgery, laparoscopy

## Abstract

**Background**: Right hemicolectomy remains the standard surgical treatment for right colon diseases. This study evaluates robotic versus laparoscopic approaches to determine optimal, minimally invasive strategies, balancing technical efficacy with healthcare economics. **Materials and Methods**: This retrospective study evaluates robotic versus laparoscopic approaches using data from 46 studies (2003–2025) involving 36,868 patients (6,312 robotic, 30,547 laparoscopic). Primary outcomes assessed were lymph node yield, operative time, and hospital stay; secondary outcomes included blood loss, conversion rates, infections, readmissions, and costs. **Results**: Concerning robotic surgery, lymph node harvest was higher (MD 1.34 for CME; MD 1.27 for non-CME). Laparoscopy showed shorter operative times (MD 25.73 for CME; MD 42.45 for non-CME). Hospital stays showed no significant difference. Robotics demonstrated lower blood loss in non-CME cases (MD -0.38). Conversion rates favored robotics (1% vs. 10% for CME; 4% vs. 8% for non-CME). No significant differences were found in wound infections or non-CME readmissions, though robotics showed lower CME readmission costs (MD 5.34). There were several study-acknowledged limitations, including surgeon preference bias, protocol variability, learning curves, and evolving techniques over the 22-year period. Cost analyses considered both direct surgical expenses and postoperative care. **Conclusions**: While robotics offers advantages in oncological resection and procedural stability, laparoscopy maintains efficiency benefits. These findings contribute to ongoing discussions about optimal, minimally invasive approaches for right colon pathologies, balancing technical efficacy with healthcare economics. By comparing surgical techniques, surgeon expertise, patient characteristics, and healthcare costs across multiple institutions, this study seeks to provide meaningful insights for surgical decision-making and further standardization.

## 1. Introduction

Colon cancer represents a major global health burden and remains one of the most frequently diagnosed malignancies worldwide [1]. Surgical resection is the cornerstone of curative treatment for right-sided colon cancer, with right hemicolectomy representing the standard surgical approach. Over the past decades, minimally invasive surgery has progressively replaced open techniques, demonstrating advantages in terms of postoperative recovery, reduced surgical trauma, and shorter hospital stay [1,2].

Among minimally invasive approaches, laparoscopic right hemicolectomy has become widely adopted due to its safety and reproducibility. However, laparoscopy is associated with intrinsic technical limitations, including two-dimensional visualization, restricted instrument articulation with limited degrees of freedom, and ergonomic challenges related to fixed pivot points and instrument collision [3]. These factors may complicate precise dissection of embryological planes and vascular structures, particularly in technically demanding procedures.

In 2009, the concept of complete mesocolic excision (CME) was introduced, adapting the principles of total mesorectal excision to colon cancer surgery. CME is based on sharp dissection along embryological planes, central vascular ligation, and intact mesocolic envelope removal, with the aim of improving oncological radicality through more extensive lymphadenectomy. Since its introduction, CME has been increasingly adopted, although its technical complexity has raised concerns regarding reproducibility and safety, especially when performed laparoscopically.

Robotic surgery has been proposed as a potential solution to overcome some limitations of conventional laparoscopy. Robotic platforms offer three-dimensional visualization, tremor filtration, enhanced dexterity through articulated instruments, and improved ergonomics for the surgeon [4]. These features may theoretically facilitate precise dissection during right hemicolectomy, particularly in CME procedures. Nevertheless, the widespread adoption of robotic surgery remains limited by higher costs, longer operative times, and variability related to surgeon experience and learning curves.

Despite the growing body of literature comparing robotic and laparoscopic right hemicolectomy, evidence regarding their relative advantages remains heterogeneous and sometimes conflicting. In particular, the impact of robotic surgery on perioperative outcomes, technical surrogate markers of oncological adequacy, and healthcare resource utilization remains debated, especially when considering CME and non-CME procedures separately.

Therefore, the aim of this systematic review and meta-analysis was to compare robotic and laparoscopic right hemicolectomy by analyzing perioperative outcomes, short-term postoperative results, and hospitalization costs, with subgroup analyses according to the use of complete mesocolic excision. By synthesizing data from studies published over a 22-year period, this work seeks to provide a comprehensive and balanced assessment of the current evidence, while acknowledging the methodological limitations inherent to the available literature.

## 2. Materials and Methods

### 2.1. Study Design and Protocol Registration

A systematic review and meta-analysis was conducted to compare robotic and laparoscopic right hemicolectomy for colon cancer. The study was designed and reported in accordance with the Preferred Reporting Items for Systematic Reviews and Meta-Analyses (PRISMA) statement (see Supplementary Materials) [5]. The methodological approach was predefined to minimize selection and reporting bias, acknowledging the expected heterogeneity related to study design, surgical era, and institutional practice. The review protocol was prospectively registered in the PROSPERO database (registration number: CRD420251040868).

### 2.2. Eligibility Criteria

Randomized controlled trials and comparative observational studies evaluating robotic versus laparoscopic right hemicolectomy for colon cancer were considered eligible. Given the limited availability of randomized controlled trials in this surgical field, the majority of included studies were retrospective or prospective observational analyses. Both procedures performed with complete mesocolic excision (CME) and without CME were included. CME was defined as sharp dissection along embryological planes with central vascular ligation and intact mesocolic envelope removal.

Studies were excluded if they were non-comparative, case reports, conference abstracts, narrative reviews, or if outcome data were incomplete or not extractable for quantitative synthesis. When overlapping patient populations were identified across multiple publications, only the most recent or most comprehensive study was included to avoid data duplication.

### 2.3. Literature Search Strategy

A comprehensive and systematic literature search was conducted to identify relevant studies published between January 2003 and March 2025. The electronic databases Medline/PubMed, Scopus, and Web of Science were searched without language restrictions. The search strategy was developed using predefined free-text terms related to the surgical approach and anatomical site, combined through Boolean operators. The same search string was applied consistently across all databases: “Robotic” AND “Laparoscopic” AND “Right” AND “Colectomy”.

The extended time frame was intentionally selected to capture the evolution of minimally invasive and robotic techniques over time, acknowledging potential variability related to surgical era, technological development, and learning curves. Reference lists of included studies were manually screened to identify additional eligible articles. Gray literature was explored through Google Scholar to minimize publication bias.

### 2.4. Study Inclusion Process

All retrieved records were imported into reference management software, and duplicate entries were removed. Two reviewers (M.S. and R.C.) independently screened titles and abstracts for eligibility. Full-text articles were assessed when abstracts met the inclusion criteria or when eligibility was unclear. Discrepancies were resolved through discussion until consensus was reached.

### 2.5. Data Extraction

Data extraction was independently performed by the same two reviewers using a predefined data collection form. Extracted variables included year of publication, country, study design, patient selection criteria, surgical technique, use of CME, sample size, demographic characteristics, and reported outcomes. When continuous data were reported as medians with ranges or interquartile ranges, standard conversion methods were applied when feasible to allow quantitative synthesis.

### 2.6. Outcomes of Interest

Primary outcomes were the number of harvested lymph nodes, operative time, and length of hospital stay. Lymph node yield was considered a technical surrogate marker of oncological adequacy rather than a direct oncological outcome. Secondary outcomes included estimated intraoperative blood loss, conversion to open surgery, surgical site infection, readmission rate, and overall hospitalization costs.

Outcomes such as anastomotic leak, reoperation, thromboembolic events, postoperative ileus, incisional hernia, extraction site, and type of anastomosis (intracorporeal versus extracorporeal) were not included due to inconsistent reporting across studies and insufficient data for reliable quantitative synthesis. Subgroup analyses were conducted according to the use of CME (CME vs. non-CME).

### 2.7. Statistical Analysis

For dichotomous variables, risk ratios (RRs) with 95% confidence intervals (CIs) were calculated. Continuous outcomes were analyzed using weighted mean differences (WMDs) when outcomes were reported using comparable units, or standardized mean differences (SMDs) when different measurement scales or reporting methods were used. The use of standardized mean difference was primarily applied to blood loss and cost analyses due to heterogeneity in reporting units and calculation methods across studies.

Meta-analyses were performed using the Mantel–Haenszel method. Given the expected clinical and methodological heterogeneity, a random-effects model was applied for all analyses. Statistical heterogeneity was assessed using the Chi-square test and quantified by the I^2^ statistic, with values above 75% indicating substantial heterogeneity. Pooled estimates associated with high or extreme heterogeneity were interpreted with caution and considered exploratory. All analyses were conducted using Review Manager (RevMan) software version 5.3.5 (The Nordic Cochrane Centre, Copenhagen, Denmark).

### 2.8. Risk of Bias Assessment

The methodological quality of non-randomized studies was evaluated using the Risk of Bias in Non-randomized Studies of Interventions (ROBINS-I) tool. Risk-of-bias judgments were classified as low, moderate, serious, or critical according to ROBINS-I guidance. Particular attention was paid to bias due to confounding, participant selection, and missing data, which were anticipated limitations of observational study designs. Graphical summaries of the risk-of-bias assessment were generated using the ROBVIS online visualization platform.

## 3. Results

### 3.1. Study Selection

**Figure 1:**
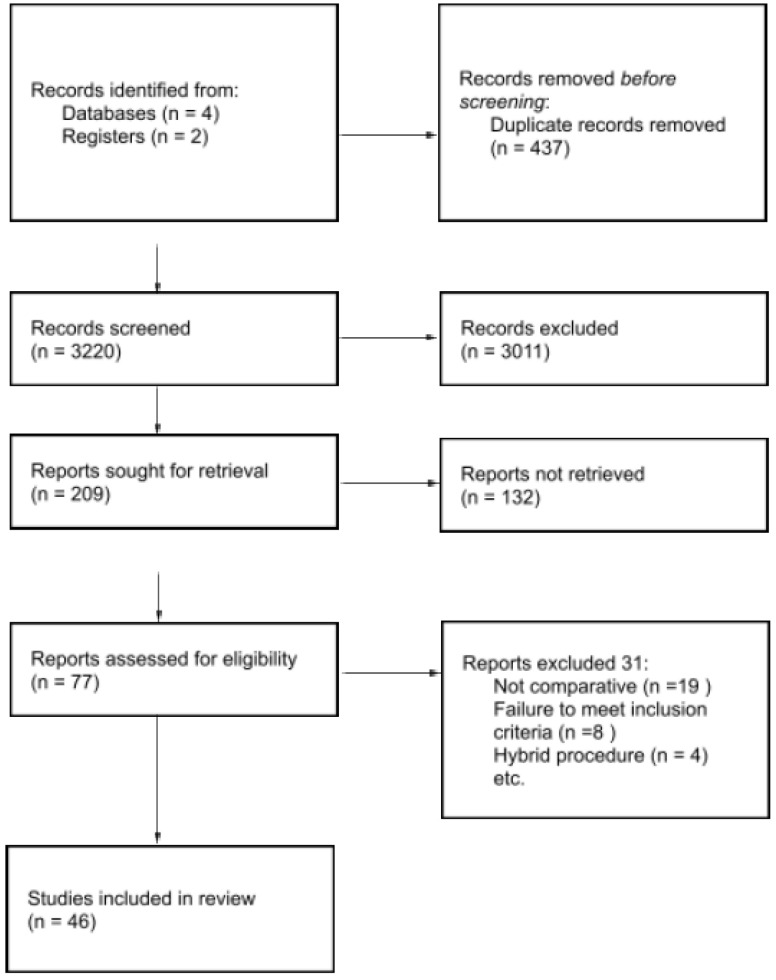
Prisma flow chart of included and excluded participants and the statistical analyses performed.

The literature search yielded 3,220 records. After removal of 437 duplicates, 2,783 records were screened by title and abstract. Of these, 2,574 were excluded for not meeting the inclusion criteria. Sixty-one full-text articles were assessed for eligibility, resulting in the exclusion of 31 studies. Ultimately, 46 studies were included in the qualitative and quantitative synthesis. The study selection process is illustrated in Figure 1.

### 3.2. Study Characteristics

The 46 included studies comprised a total of 36,868 patients, of whom 6,312 underwent robotic surgery, and 30,547 underwent laparoscopic surgery. Most studies were conducted in Europe (45.7%), followed by Asia (21.7%) and other regions, including the United States, Russia, and Australia. Publication years ranged from 2003 to 2025. Detailed study characteristics and patient demographics are summarized in Table 1 and Table 2.

### 3.3. Risk of Bias Within Studies

**Figure 2:**
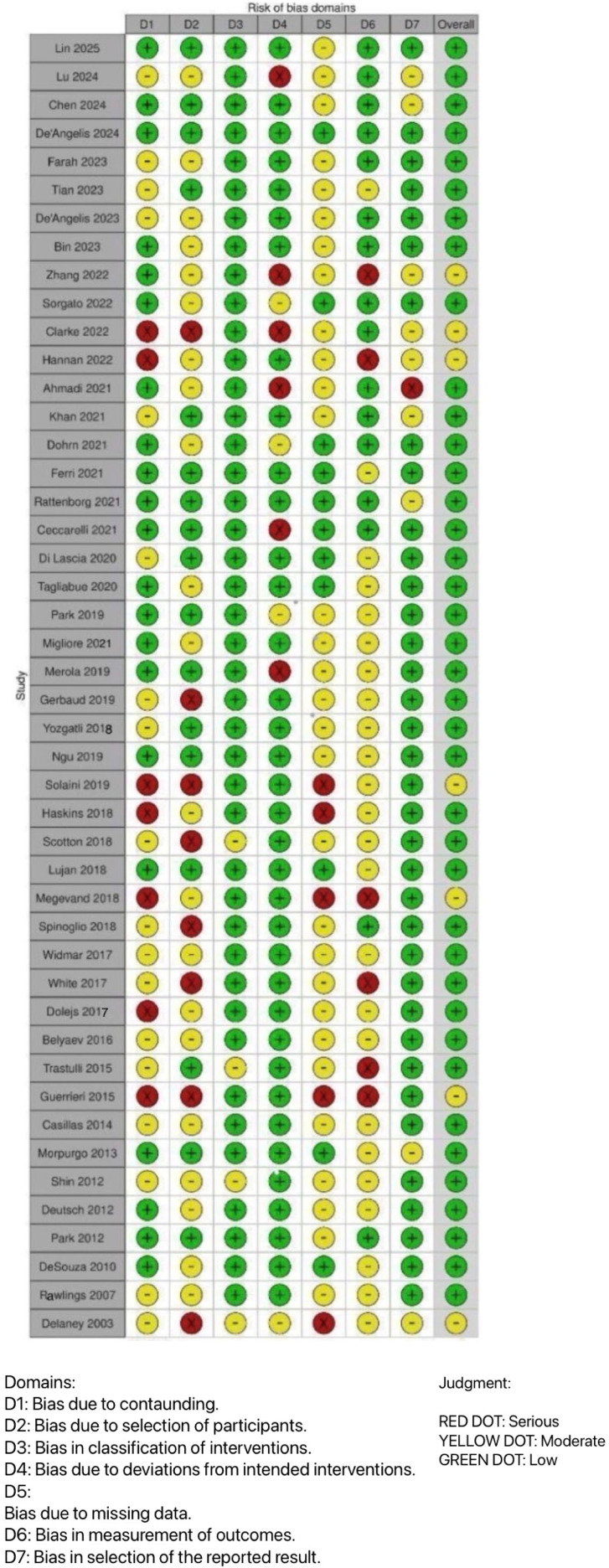
The risk of bias for each of the 7 categories due to confounding data, selection of participants, classification of intervention, deviation from intended interventions, missing data, outcomes measurements, and reported results [1,2,3,4,5,6,7,8,9,10,11,12,13,14,15,16,17,18,19,20,21,22,23,24,25,26,27,28,29,30,31,32,33,34,35,36,37,38,39,40,41,42,43,44,45,46]. The green stands for no risk, yellow for moderate, and red for high risk of bias.

According to the ROBINS-I assessment, the methodological quality of included studies was variable. Most studies were judged to have low to moderate risk of bias overall, while several demonstrated moderate or high risk in specific domains, particularly confounding, participant selection, and missing data. No study was classified as having a critical risk of bias. A comprehensive overview of the risk-of-bias assessment is presented in Figure 2.

### 3.4. Description of Studies

A total of 46 studies involving 36,868 patients (6,312 robotic and 30,547 laparoscopic) were included. The majority of studies (21 out of 46, 45.7%) were conducted in Europe, enrolling 4,321 patients (12%), with Italy contributing the highest number (15 studies). A total of 10 studies (21.7%) were performed in Asia, comprising 9,081 patients (24.6%). The remaining 14 studies (30.4%) were distributed as follows: 12 in the USA, 1 in Russia, and 1 in Australia. All included studies were published between 2003 and 2025. A detailed description of study characteristics and patient demographics is provided in Table 1.

### 3.5. Assessment of Risk of Bias in Included Studies

The risk of bias in the included studies was independently assessed by two authors (RC, MS).

According to the ROBINS-I tool, risk-of-bias judgment may be identified as low, moderate, serious, or critical. In total, 39 out of 46 studies were assessed as low risk of overall bias, while 7 were determined to have a moderate risk, and none had a high risk. Regarding bias due to confounding, 21 out of 46 studies were evaluated as having a low risk. Additionally, 17 studies were reported to have a moderate risk, and 7 had a high risk of bias due to confounding. In the analysis of the selection bias of study participants, 17 out of 46 reviews were evaluated as low risk and 8 as high risk. Regarding bias in the classification of the interventions, all studies had a low risk apart from five, which had a moderate risk. The risk of bias due to deviation from intended interventions was moderate in five studies and high in six studies. The evaluation of missing data bias reported that 11 studies had a low risk, 5 had a high risk, and the remaining had a moderate risk. Regarding bias in the selection of the measurement of the outcomes, 15 out of the 46 studies had a low risk, and 6 had a high risk. Additionally, for bias in the reported results, all studies were assessed as having a low risk, apart from seven that had a moderate risk and one that had a high risk, as shown in Figure 1.

## 4. Primary Outcomes

### 4.1. Retrieved Lymphatic Nodes

**Figure 3:**
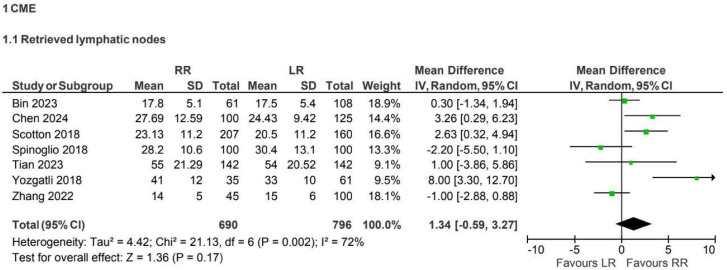
Collected data related to retrieved lymphatic nodes for CME intervention [3,5,8,9,25,29,32].

Seven retrospective observational studies [3,5,8,9,25,29,32] reported lymph node yield for CME procedures (1,486 patients: 690 robotic vs. 796 laparoscopic). The overall number of harvested lymph nodes was numerically higher in the robotic group (MD 1.34, 95% CI −0.59 to 3.27), although this difference was not statistically significant. Heterogeneity was substantial (I^2^ = 72%) (Figure 3).

**Figure 4:**
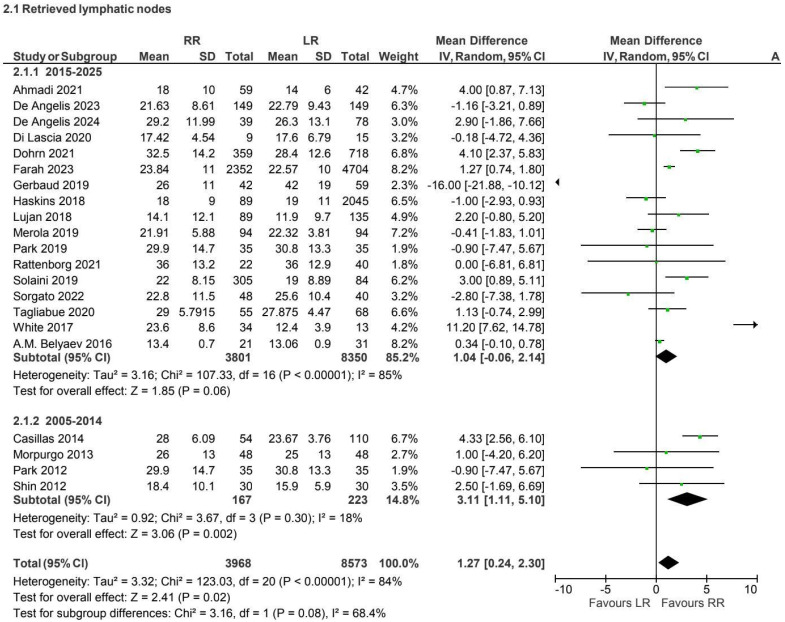
Collected data related to retrieved lymphatic nodes for non-CME intervention [4,6,7,10,13,15,17,19,20,21,23,24,27,28,30,34,36,39,40,41,43].

In the non-CME subgroup, seventeen retrospective and prospective studies [4,6,7,10,13,15,17,19,20,21,23,24,27,28,30,34,36,39,40,41,43] have been reported on lymph node retrieval. Robotic surgery was associated with a statistically significant increase in lymph node yield compared with laparoscopy; however, heterogeneity was high (I^2^ = 84%) (Figure 4).

### 4.2. Operation Time

**Figure 5:**
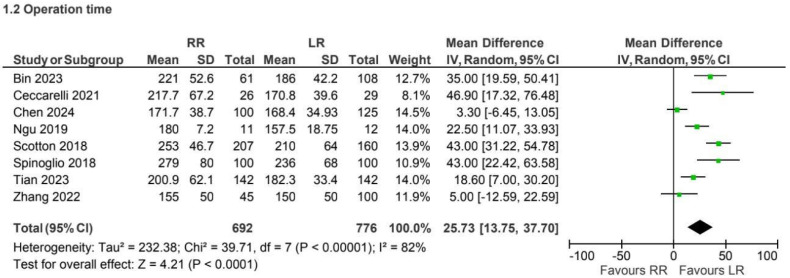
Collected data related to OP for CME intervention [3,5,8,9,18,25,26,32].

A total of 8 retrospective observational studies [3,5,8,9,18,25,26,32] reported operative times for 1468 patients in the CME group. Operative time was longer in the robotic group compared with laparoscopy (MD 25.73, 95% CI 13.75 to 37.70), with substantial heterogeneity (I^2^ = 82%) (Figure 5).

**Figure 6:**
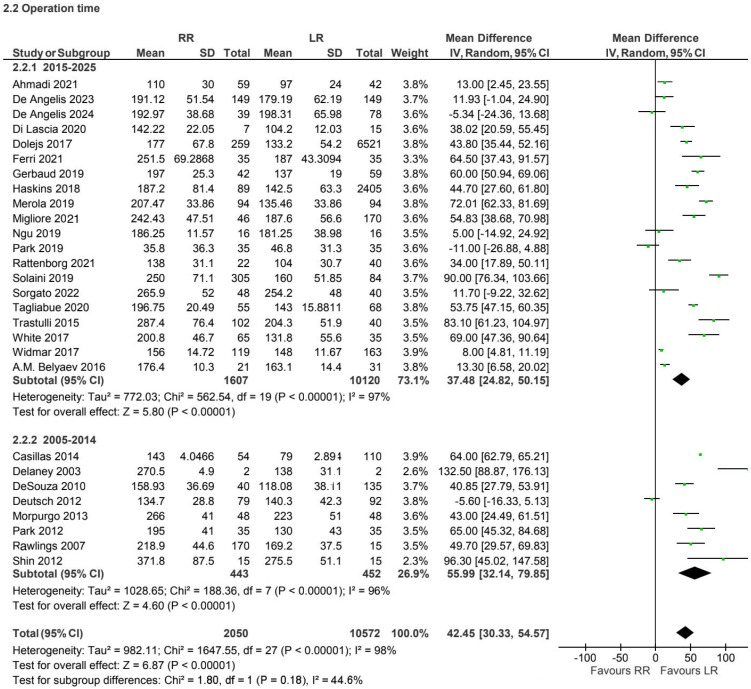
Collected data related to OP for non-CME intervention [6,7,10,13,16,17,19,20,21,22,23,24,26,27,28,33,34,35,36,37,39,40,41,42,43,44,45,46].

A total of 28 retrospective and prospective observational studies [6,7,10,13,16,17,19,20,21,22,23,24,26,27,28,33,34,35,36,37,40,41,42,43,44,45,46] reported operative time for 12,622 patients in the non-CME group. Operative time was longer in the robotic group compared with laparoscopy (MD 42.45, 95% CI 30.33 to 54.57), with considerable heterogeneity (I^2^ = 98%) (Figure 6).

### 4.3. Length of Hospital Stays

**Figure 7:**
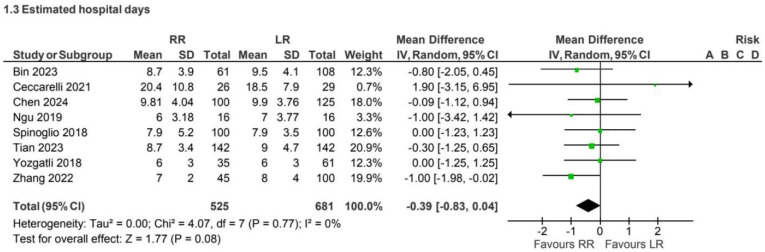
Collected data related to hospital stay for CME intervention [3,8,9,18,25,26,29,32].

A total of 8 retrospective observational studies [3,8,9,18,25,26,29,32] reported the length of hospital stay for 1,206 patients undergoing CME. Hospital stays showed no clinically meaningful difference between approaches in this subgroup (Figure 7).

**Figure 8:**
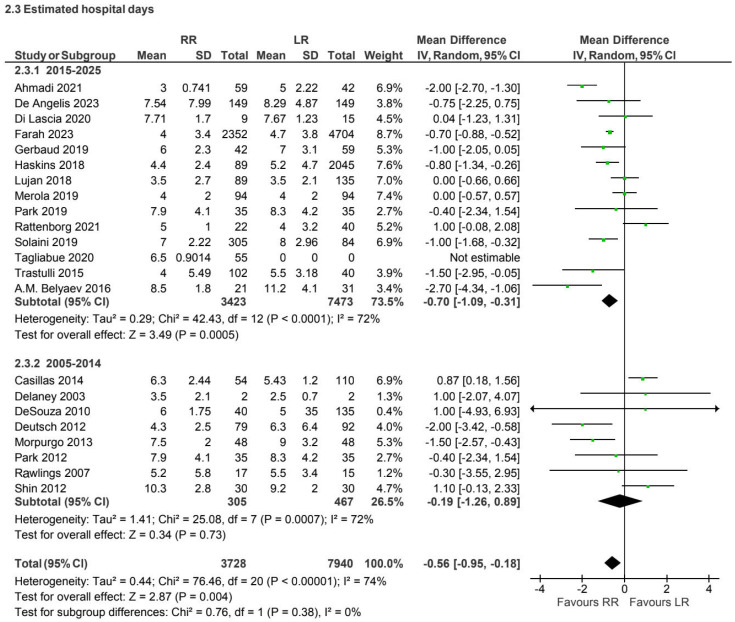
Collected data related to hospital stay for non-CME intervention [4,7,13,17,19,20,21,23,24,27,28,30,36,37,39,40,41,42,43,44,45,46].

A total of 22 prospective and retrospective studies [4,7,13,17,19,20,21,23,24,27,28,30,36,37,39,40,41,42,43,44,45,46] involving 11,668 patients reported the length of hospital stay for non-CME procedures. A trend toward shorter hospitalization with robotic surgery was observed in more recent studies; overall heterogeneity was moderate (I^2^ = 74%) (Figure 8).

## 5. Secondary Outcomes

### 5.1. Estimated Blood Loss

**Figure 9:**
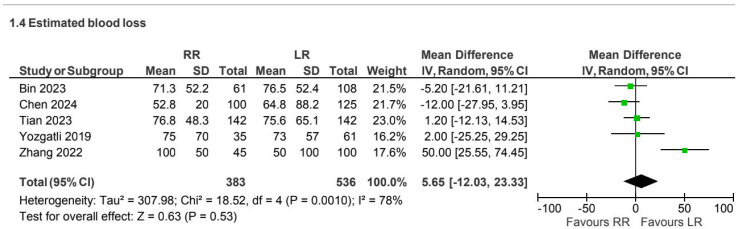
Collected data related to blood loss for CME intervention [8,9,18,25,29].

Five retrospective observational studies [8,9,18,25,29] reported estimated blood loss for the CME subgroup (919 patients). No statistically significant difference was observed between approaches (MD 5.65, 95% CI −12.03 to 23.33), with substantial heterogeneity (I^2^ = 78%) (Figure 9).

**Figure 10:**
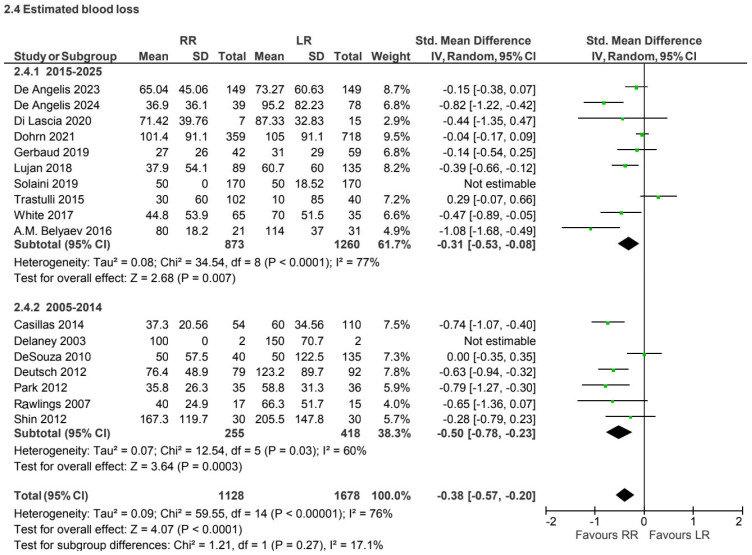
Collected data related to blood loss for non-CME intervention [6,7,15,19,24,27,30,34,36,37,39,41,42,43,44,45,46].

Fifteen prospective and retrospective studies [6,7,15,19,24,27,30,34,36,37,39,41,42,43,44,45,46] reported blood loss for the non-CME subgroup (2,806 patients). Robotic surgery was associated with lower estimated blood loss (SMD −0.38, 95% CI −0.57 to −0.20), with high heterogeneity (I^2^ = 76%) (Figure 10).

### 5.2. Conversion from Minimally Invasive to Open Right Hemicolectomy

**Figure 11:**
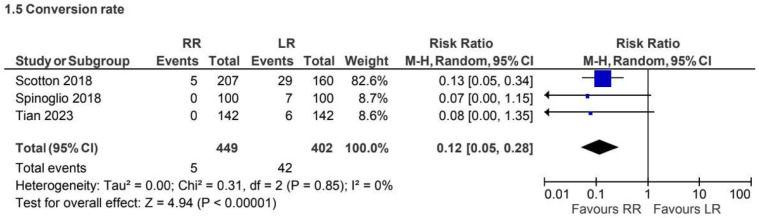
Collected data related to conversion rate for CME intervention [5,29,32].

A total of 3 retrospective observational studies [5,29,32] including 851 patients, reported conversion rates for the CME subgroup. Conversion occurred in 1% versus 10% of cases in the robotic and laparoscopic groups, respectively. Robotic surgery was associated with a lower conversion rate (RR 0.12, 95% CI 0.05 to 0.28) (Figure 11).

**Figure 12:**
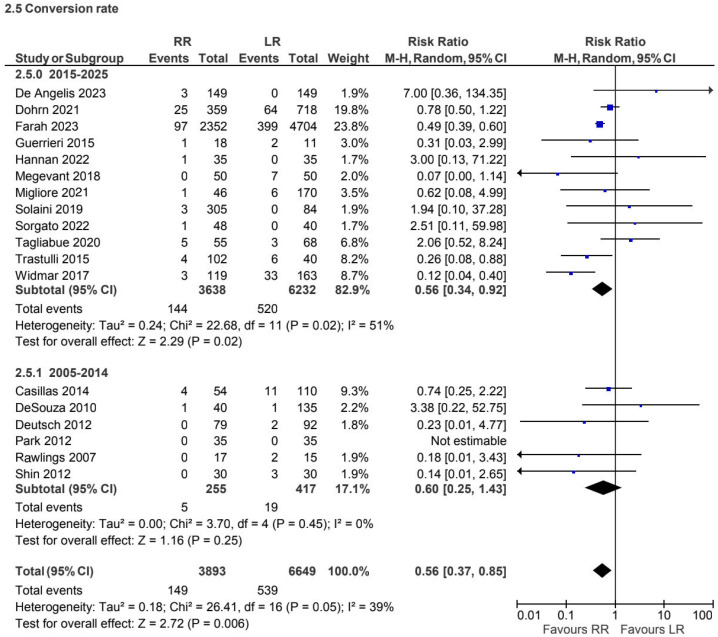
Collected data related to conversion rate for non-CME intervention [4,7,10,12,15,20,22,27,31,33,37,38,39,41,42,43,44,45].

A total of 18 retrospective and prospective studies [4,7,10,12,15,20,22,27,31,33,37,38,39,41,42,43,44,45], including 10,542 patients, reported conversion rates for the non-CME subgroup. Robotic surgery was associated with a lower conversion rate compared with laparoscopy (RR 0.56, 95% CI 0.37 to 0.85), with low heterogeneity (I^2^ = 39%) (Figure 12).

### 5.3. Wound Infection

**Figure 13:**
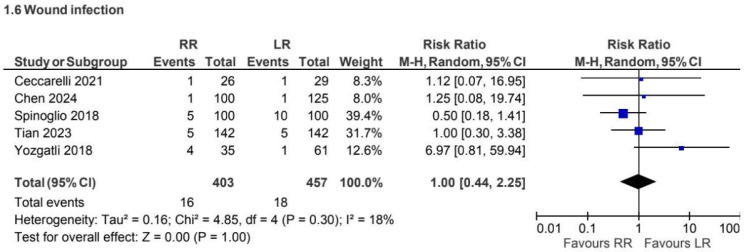
Collected data related to wound infection for CME intervention [3,5,18,25,32].

A total of 5 retrospective observational studies [3,5,18,25,32], including 860 patients, reported wound infection for CME procedures. No statistically significant difference was observed between approaches (RR 1.00, 95% CI 0.44 to 2.25), with low heterogeneity (I^2^ = 18%) (Figure 13).

**Figure 14:**
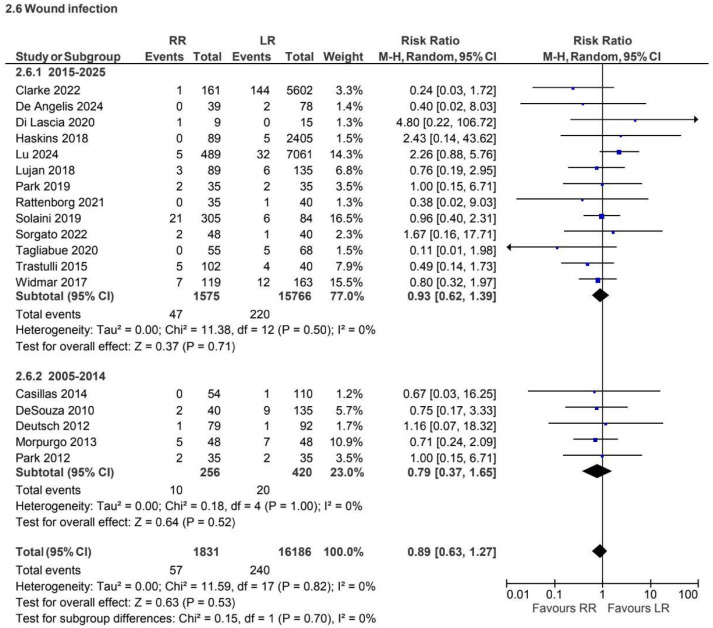
Collected data related to wound infection for non-CME intervention [2,6,10,11,17,19,20,21,27,28,30,33,37,39,40,42,43,44].

A total of 18 retrospective and prospective studies [2,6,10,11,17,19,20,21,27,28,30,33,37,39,40,42,43,44], including 18,017 patients, reported wound infection for non-CME procedures. No statistically significant difference was observed between approaches (RR 0.89, 95% CI 0.63 to 1.27) (Figure 14).

### 5.4. Readmission Rate

**Figure 15:**
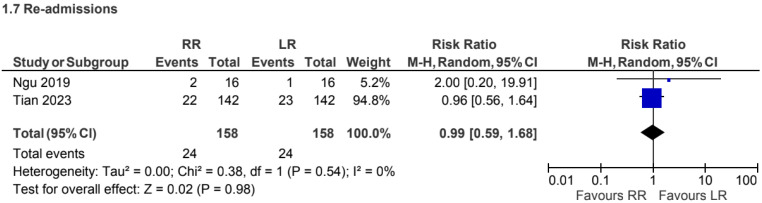
Collected data related to readmission rate for CME intervention [5,26].

A total of 2 retrospective observational studies [5,26], including 316 patients, reported readmission rates for CME procedures. No statistically significant difference was observed between robotic and laparoscopic approaches (RR 0.99, 95% CI 0.59 to 1.68) (Figure 15).

**Figure 16:**
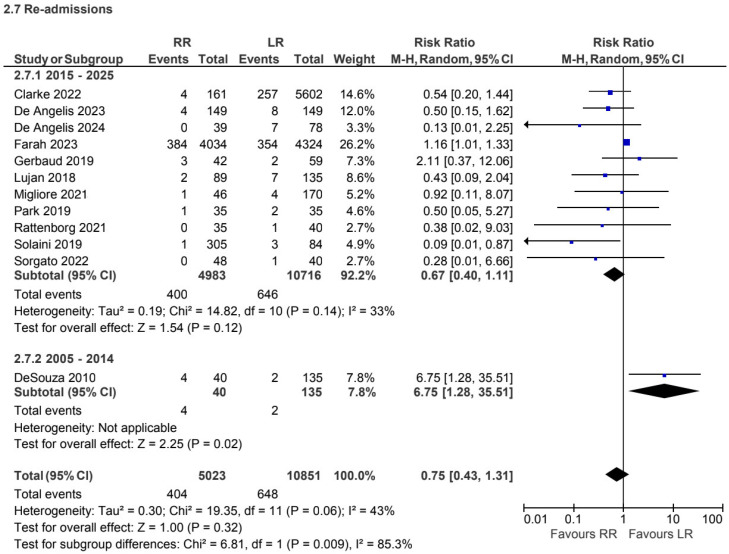
Collected data related to readmission rate for non-CME intervention [4,6,7,10,11,17,21,22,24,27,30,44].

A total of 12 retrospective and prospective studies [4,6,7,10,11,17,21,22,24,27,30,44], including 15,874 patients, reported readmission rates for non-CME procedures. No statistically significant difference was observed between robotic and laparoscopic approaches (RR 0.75, 95% CI 0.43 to 1.31) (Figure 16).

### 5.5. Hospitalization Cost (Reported in US Dollars)

**Figure 17:**
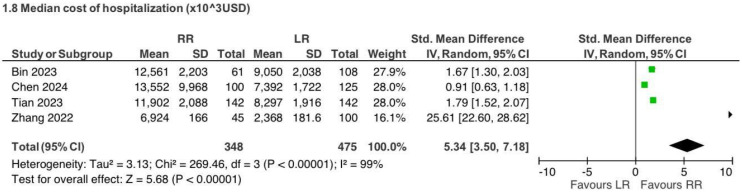
Collected data related to the median cost of the whole hospitalization period for CME intervention [3,5,8,9].

Four retrospective observational studies [3,5,8,9] reported hospitalization costs for CME procedures (823 patients). Robotic surgery was associated with higher hospitalization costs compared with laparoscopy (SMD 5.34, 95% CI 3.50 to 7.18), with extreme heterogeneity (I^2^ = 99%) (Figure 17).

**Figure 18:**
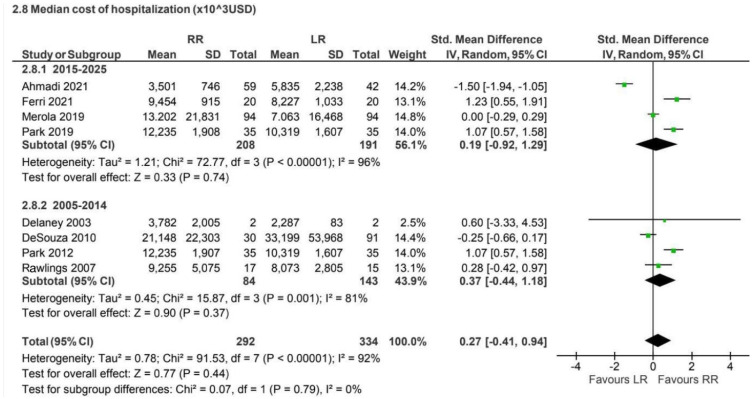
Collected data related to the median cost of the whole hospitalization period for non-CME intervention [13,16,21,23,43,44,45,46].

Eight retrospective and prospective studies [13,16,21,23,43,44,45,46] reported hospitalization costs for non-CME procedures (626 patients). No statistically significant difference was observed between approaches (SMD 0.27, 95% CI −0.41 to 0.94), with substantial heterogeneity (I^2^ = 92%) (Figure 18).

## 6. Discussion

Right-sided colon cancer surgery remains technically demanding due to the anatomical complexity of mesocolic planes, vascular variability, and lymphatic drainage patterns. The introduction of complete mesocolic excision (CME), aimed to improve oncological radicality by applying the principles of embryological plane dissection to colon cancer surgery. Over the past two decades, minimally invasive approaches have progressively replaced open surgery, with laparoscopic right hemicolectomy becoming widely adopted as a standard technique [4,5].

More recently, robotic platforms have been introduced with the aim of overcoming some intrinsic limitations of laparoscopy, including restricted instrument articulation, two-dimensional visualization, and ergonomic constraints [5,16]. The present systematic review and meta-analysis synthesized data from 46 comparative studies published between 2003 and 2025, including more than 36,000 patients, to evaluate perioperative and short-term outcomes of robotic versus laparoscopic right hemicolectomy, stratified by CME and non-CME techniques. Given the observational nature of most included studies and the substantial heterogeneity observed across several outcomes, the findings should be interpreted with appropriate caution.

Lymph node yield represents a technical surrogate marker of oncological adequacy rather than a direct measure of oncological effectiveness. In this analysis, robotic surgery was associated with a modest increase in the number of retrieved lymph nodes, particularly in non-CME procedures [6,7,10,13,17,19,20,21,23,24,27,28,30,34,36]. However, the absolute differences were small and characterized by high heterogeneity, suggesting that factors such as surgeon experience, learning-curve effects, institutional protocols, and patient selection may have contributed substantially to these findings. Importantly, no conclusions regarding oncological superiority can be drawn in the absence of long-term outcomes such as disease-free or overall survival, which were not consistently reported across the included studies.

Operative time consistently favored the laparoscopic approach in both CME and non-CME subgroups [3,5,8,9,18,26,29,32]. This finding remained stable across different publication periods and likely reflects the additional setup time, docking procedures, and technical complexity associated with robotic surgery, particularly during earlier phases of adoption. While operative duration is an important efficiency metric, its clinical relevance should be interpreted in the context of overall perioperative outcomes and institutional expertise.

Length of hospital stay showed no clinically meaningful differences between approaches in most analyses, although a trend toward shorter hospitalization with robotic surgery was observed in more recent non-CME studies [6,7,13,17,19,20,21,23,24,27,28,30,36]. This temporal pattern may reflect increasing familiarity with robotic platforms, improvements in perioperative care, and enhanced recovery protocols rather than an intrinsic advantage of the surgical approach itself.

Among secondary outcomes, robotic surgery demonstrated lower conversion rates to open surgery in both CME and non-CME procedures [5,29,32]. This finding may be related to improved visualization, instrument articulation, and ergonomic advantages offered by robotic systems, particularly in technically challenging cases. Estimated blood loss was generally low in both approaches, with a modest advantage for robotic surgery in non-CME procedures [6,7,15,19,24,27,30,34,36]; however, variability in reporting methods and measurement techniques limits the interpretability of this outcome.

No significant differences were observed in wound infection or readmission rates between robotic and laparoscopic approaches [3,5,18,25,32]. It should be noted that definitions of surgical site infection varied across studies, and important postoperative complications such as anastomotic leak, reoperation, thromboembolic events, postoperative ileus, and incisional hernia could not be analyzed due to inconsistent reporting. Similarly, data regarding anastomotic technique (intracorporeal versus extracorporeal), extraction site, patient comorbidities, and neoadjuvant treatments were insufficient for meaningful quantitative synthesis.

Cost analysis represented one of the most heterogeneous outcomes, reflecting differences in healthcare systems, reimbursement models, institutional accounting methods, and the long study period considered [3,5,8,9,13,16,21,23,43,44,45,46]. While laparoscopic surgery was generally associated with lower costs, particularly in CME procedures, these findings should be regarded as exploratory and not directly comparable across settings.

Overall, the substantial heterogeneity observed across multiple outcomes underscores the influence of non-technical factors, including study design, geographic region, surgeon expertise, and temporal evolution of surgical technology. These elements limit the strength of pooled estimates and highlight the need for cautious interpretation.

## 7. Conclusions

This systematic review and meta-analysis suggest that both robotic and laparoscopic right hemicolectomy are safe and effective minimally invasive approaches, each associated with distinct technical and perioperative characteristics. Robotic surgery may offer advantages in terms of conversion rates and selected technical surrogate markers, while laparoscopic surgery remains more efficient with respect to operative time and overall costs.

However, the observed differences are generally modest and characterized by substantial heterogeneity, limiting definitive conclusions regarding clinical or oncological superiority. In the absence of robust long-term oncological outcomes and standardized reporting of key postoperative complications, surgical approach selection should be guided by surgeon expertise, institutional resources, patient characteristics, and procedural complexity rather than by expectations of inherent superiority.

Future prospective studies and randomized trials with standardized outcome definitions, long-term follow-up, and detailed reporting of perioperative variables are needed to better define the role of robotic surgery in right-sided colon cancer and to clarify its potential benefits within contemporary surgical practice.

## Figures and Tables

**Table 1:** The complete list of included studies organized by year and listing nationality, year of publication, study period, type of study, surgical modality, CME or non-CME, number of patients included, gender of the population, mean age, and mean BMI.

Author	Nation	Y.O. P	Study Period	Type of Study	Surgical Type	Type of Robot	Feature	Sample Size (RR/LR)	Gender (M:F) RR/LR	Mean Age (RR/LR)	BMI (Mean kg/m^2^ RR/RL)
Lin	Taiwan	2025	2018–2024	ROS	RR LR-2D	NA	No CME	50/241	24:26/117:124	72.9/67.7	24.2/24
Lu	Taiwan	2024	2005/2018	ROS	RR LR-2D	NA	No CME	489/7061	185:304/2728:4333	84.3/84.8	NA
Chen	China	2024	2019–2022	ROS	RR LR-2D	Da Vinci robotic surgical system	CME	100/125	NA	67.1/63.6	22.6/23.07
De’Angelis	Italy	2024	2014–2020	ROS	RR LR-2D	Da Vinci robotic surgical system	No CME	45/155	NA	76/72	26.78/26.4
Farah	USA	2023	2015–2020	ROS	RR LR-2D	NA	No CME	2352/4704	1106:1246/6907:7723	66.7/68	27.9/27.4
Tian	China	2023	2016–2021	ROS	RR LR-2D	da Vinci Si	CME	149/233	81:68/123/110	64.1/64.4	22.5/22.3
De’Angelis	Italy	2023	2014–2020	ROS	RR LR-2D	Da Vinci robotic surgical system	No CME	194/402	NA	70.7/71.3	27.2/26.7
Bin	China	2023	2016–2021	ROS	RR LR-2D	Da Vinci robotic surgical system	CME	61/108	33:28/56:52	61/62	22.9/22.4
Zhang	China	2022	2016–2018	ROS	RR LR-2D	NA	CME	46/186	19:27/87:95	NA	NA
Sorgato	Italy	2022	2018–2019	POS	RR LR-2D	Da Vinci Si HD™	No CME	48/40	27:21/28:12	71/68	25.5/26.6
Clarke	Australia	2022	2007–2020	ROS	RR LR-2D	NA	No CME	161/5602	NA	73.4/72.1	NA
Hannan	USA	2022	2016–2020	ROS	RR LR-2D	Da Vinci Xi	No CME	35/35	18:17/18/17	66.5/69.7	NA
Ahmadi	Australia	2021	2015–2018	ROS	RR LR-2D	Da Vinci Si	No CME	59/42	NA	75/75	27/27
Khan	United Kingdom	2021	2014–2017	ROS	RR LR-2D	Da Vinci VR Si/X	CME	40/80	19:21/37:43	69/71	26/28
Dohrn	Denmark	2021	2015–2018	ROS	RR LR-2D	NA	No CME	359/718	181:178/378:340	73.3/73.7	25.9/25.6
Ferri	Spain	2021	2013–2017	POS	RR LR-2D	Da Vinci Si/Da Vinci Xi	No CME	35/35	23:12/20:15	67/68	23/25
Rattenborg	Denmark	2021	2015/2018	POS	RR LR-2D	Da Vinci Xi	No CME	22/40	9:13/15:25	71/73	25.5/25.5
Ceccarelli	Italy	2021	2014–2019	ROS	RR LR-3D	Da Vinci Si/Da Vinci Xi	CME	20/20	14:6/13:7	70.6/74.6	23/24.1
Migliore	Italy	2021	2010–2018	ROS	RR LR-2D	e da Vinci^®^ Si™ System	No CME	46/170	NA	69/72	26/25.5
Di Lascia	Italy	2020	2014–2017	ROS	RR LR-2D	Da Vinci Xi	No CME	7/15	3:4/7:8	76.2/74.5	27.75/27.5
Tagliabue	Italy	2020	2014–2019	ROS	RR LR-2D	NA	No CME	55/68	NA	72/71.8	24.6/25.38
Park	Korea	2019	2009–2011	PRO	RR LR-2D	Da Vinci Si HD™	No CME	35/35	14:21/16:19	62.8/66.5	24.4/23.8
Merola	Italy	2019	2012–2017	ROS	RR LR-2D	Da Vinci Si/Da Vinci Xi	No CME	94/94	60:34/61:33	69.4/72	26.9/27.9
Gerbaud	France	2019	2013–2019	ROS	RR LR-2D	NA	No CME	42/59	21:21/31:28	67/72	26/24
Ngu	Singapore	2019	2015–2017	ROS	RR LR-2D	Da Vinci Xi	CME	16/16	68.6/69.6	10:6/6:10	23.7/24.7
Solaini	Italy	2019	2007–2017	ROS	RR LR-2D	NA	No CME	305/84	39:45/163:142	NA	NA
Haskins	USA	2018	2021–2014	ROS	RR LR-2D	NA	No CME	89/2405	49:40/1129:1276	68.9/68.3	29.3/28.5
Yozgatli	Turkey	2018	2015–2017	ROS	RR LR-2D	Da Vinci Xi	CME	35/61	NA	65/65	29/27
Scotton	Italy	2018	1998–2007	ROS	RR LR-2D	Da Vinci Xi	CME	206/160	108:98/80:80	70.1/70.3	26/25.6
Lujan	USA	2018	2009–2015	ROS	RR LR-2D	Da Vinci Si/Xi	No CME	89/135	48:41/61:74	70.9/72.6	28.4/27.1
Megevand	Italy	2018	2010–2015	ROS	RR LR-2D	DaVinci^®^ System	No CME	50/50	28:22/24:26	70.3/69.6	26.2/25.2
Spinoglio	Italy	2018	2005–2015	ROS	RR LR-2D	Da Vinci Si	CME	100/100	44:54/54:47	71.2/71.2	25.1/25.8
Widmar	USA	2017	2009–2014	ROS	RR LR-2D	NA	No CME	119/163	64:55/83:80	68/64	28/29
White	USA	2017	2005–2017	ROS	RR LR-2D	Da Vinci Si/Da Vinci Xi	No cME	65/35	36:29/18/17	63/67	28/27.6
Dolejs	USA	2017	2012–2014	ROS	RR LR-2D	NA	No CME	259/6521	138:121/3482:3039	65.3/53.7	24.5/23.8
A.M. Belyaev	Russia	2016	2013–2015	ROS	RR LR-2D	NA	No CME	21/31	5:16/8:23	69.8/70.6	26.2/23.7
Trastulli	Italy	2015	2005–2014	ROS	RR LR-2D	Da Vinci Si HD™	No CME	102/40	56:46/25:15	68.8/71.5	25.6/26.6
Guerrieri	Italy	2015	2013–2014	ROS	RR LR-3D	NA	No CME	18/11	NA	71/66	26/26

**Table 2:** Studies from 2003 to 2014.

Author	Nation	Y.O.P	Study Period	Type of Study	Surgical Type	Type of Robot	Feature	Sample Size (RR/LR)	Gender (M:F) RR/LR	Mean Age (RR/LR)	BMI (Mean kg/m^2^ RR/RL)
Casillas	USA	2014	2005–2012	ROS POS	RR LR-2D	NA	No CME	52/110	NA	65/71	26.9/27
Morpurgo	Italy	2013	2009–2012	CC	RR LR-2D	Da Vinci Robotic system	No CME	48/48	27:21/16:32	68/74	25/28
Shin	Korea	2012	2010–2011	PRO	RR LR-2D	Da Vinci SH	No CME	30/30	18:12/18:12	58/63	26.4/26
Deutsch	USA	2012	2004–2009	ROS	RR LR-2D	Da Vinci Robotic system	No CME	79/92	12:6/25:22	65/71	25/28
Park	Korea	2012	2009–2011	RCT	RR LR-2D	NA	No CME	35/35	14:21/16:19	63/67	24.4/23.8
DeSouza	USA	2010	2005–2009	ROS	RR LR-2D	NA	No CME	40/135	22:18/62:73	71.3/65.3	27.3/26.6
Rawlings	USA	2007	2002–2005	ROS	RR LR-2D	Da Vinci Robotic system	No CME	17/15	8:9/6:9	65/63	26/28
Dalaney	USA	2003	2001/2002	POS	RR LR-2D	Da Vinci Robotic system	No CME	2/2	1:1/1:1	63/64.5	31.5/25

ROS: Retrospective observational study; POS: Prospective observational study; RCT: Randomized Control Trial; CC: Case Control; RR: Robotic Surgery; LR 2D/3D: Laparoscopic Surgery; NA: Not available.

## Data Availability

No new data were generated or analyzed in this study. Data sharing is not applicable.

## Supplementary Materials

The following supporting information can be downloaded at: https://www.mdpi.com/article/10.3390/jcm15041493/s1, PRISMA 2020 Checklist.
